# Indications for Operation in Ectopic Gestation

**Published:** 1890-12

**Authors:** Charles A. L. Reed

**Affiliations:** Cincinnati, O.; Surgeon to the Cincinnati Free Hospital for Women, Professor of Gynecology and Abdominal Surgery at the Cincinnati College of Medicine and Surgery. Fellow of the American Association of Obstretricans and Gynecologists. Chairman of Lecturers on Obstretrics and Diseases of Women of the American Medical Association


For Daniel's Texas Medical Journal.

### SYNOPSIS OF A PAPER ON INDICATIONS FOR OPERATION IN ECTOPIC GESTATION.

BY CHARLES A. L. REED, M. D., CINCINNATI, O.

Surgeon to the Cincinnati Free Hospital for Women, Professor of Gynecology and Abdominal Surgery at the Cincinnati College of Medicine and Surgery. Fellow of the American Association of Obstretricans and Gynecologists. Chairman of Lecturers on Obstretrics and Diseases of Women of the American Medical Association.

Read before the Southern Surgical and Gynecological Association, at Atlanta, Georgia, November 12, 1890.

THIS paper starts with the assumption that the only proper treatment of ectopic gestation is by laparotomy or, more properly, cœliotormy. While the profession has become practically unanimous that this is the proper line of treatment, the indications for operation have been less definitely decided upon. This conviction is forced upon the observer, not only by a study of the literature of the subject, but by encountering cases which have been advised against operation by their attending physicians, until hemorrhage within the pelvis has threatened a fatality, which is but too frequently realized. The most legitimate excuse for this dilatory practice, is to be found in the confusion which has arisen with regard to the supposed uniform causal relationship of ruptured ectopic gestation to pelvic hæmatocele, and the division of the latter into "primary" and "secondary" rupture. These terms are unfortunate, and. as used in this connection, may be entirely arbitrary. "Primary" rupture is made to mean rupture beneath the peritoneum, instead of "first" rupture, as the etymology of the word would imply, while "secondary" rupture is made to mean rupture within the peritoneum, instead of "second" rupture. Whereas, an intra-peritoneal rupture may be, and frequently is a primary rupture, when spoken of with reference to the sequence of events in ectopic gestation. There would be no serious confusion even here if we were not also taught to leave extra-peritoneal hæmatoceles alone to be taken care of by absorption, and if we did not add that, as these hæmatoceles are generally caused by ruptured ectopic gestation sac, we are to relegate these cases also to the expectant plan of treatment. This conclusion is without warrant, and is responsible for hundreds of deaths annually from this one cause.

The treatment of ectopic gestation premises the diagnosis of this condition. This is obviously difficult, and in the majority of instances cannot be arrived at at all, or, if at all, only presumptively; but in all these cases conditions can be found in the pelvis, which if not conclusive of extra-uterine pregnancy, yet constitute conclusive indications for exploratory operation. The presumption of ectopic pregnancy can be arrived at before rupture, chiefly by a history of previous sterility, by a previous amenorrhœa, followed after a few weeks by irregular hemorrhage, by increase tumefaction to either side or back of the uterus, and by the existence of false decidua within the uterus. The latter fact may be safely determined by the judicious use of the Emmet curette forceps. The diagnosis after rupture is essentially the diagnosis of internal hemorrhage. Time wasted either to determine the cause of that hemorrhage, or to find out if it be "primary" or "secondary," is criminal. The thing to do is to cut down and operate. The position has been taken that time should be taken for the patient to rally from the shock. One of my own cases died simply because I waited twelve hours for reaction—a lesson which taught me the fallacy of the old teaching, and which has since saved lives at my hands. The best way to overcome shock from internal hemorrhage is to stimulate the patient by giving ether, stop the drain by ligating the bleeding vessels, and rouse the nervous system by washing out the belly with hot water.

In cases which come under observation after reaction from primary shock, shall we wait for evidences of so-called secondary rupture? In one of my cases suppuration occurred, and in another the blood clot grew until in the course of some weeks it measured nine pints when I removed it, and still another case emphasized by the latter fact. These cases all recovered, but they taught me the fallacy of waiting for absorption.

What shall be done with the appendages on the other side? In view of the fact that tubal pregnancy generally depends upon desquamative salpingitis, as confirmed by the recent observations of Formas before the American Association of Obstetricians and Gynecologists, and in view of the fact that this disease is almost uniformly bilateral, the question is at once raised: Is the woman liable to an ectopic pregnancy on the other side. Herman in the *British Medical Journal*, September 27, 1890, reports such a case; and Tait reports another with death from rupture of the second conception. Leopold Meyer reports another, and refers to verified cases by Veit and Olshausen. There are now at least ten cases on record. From this I conclude that if the patient's condition at the time of operation is such as to justify further interference, the appendages from both sides should be removed.

In the presence of rupture, the indications for operation are so imperative that no time should be lost in unnecessary preparation. In this connection, a recently reported case by Manly, of New York, offers points for the severest criticism. In that case a night was waited for a reaction, which was not realized, and a part of the day was squandered in washing the wall-paper to meet listerian indications while the woman was bleeding into her abdominal cavity, and although by dint of marvelous vitality she recovered, the lesson is none the less impressive that the delay was culpable.

The question of viability of the child has some bearing upon the line of treatment to be adopted. If the case has passed beyond the sixth month, let it proceed to term, but only under the strictest possible surveillance and with preparations at hand to operate at any moment. The statement by Manly that "ectopic, or extra-uterine pregnancy is not attended with great peril to the mother's life," [*Internat. Jour. Surgery*, October, 1890,] could be accounted absurd, if its influence were not murderous.

The conclusion to be arrived at from the most careful study of the subject, is that so clearly expressed by Tait, viz.: "If I ever should make a diagnosis of tubal pregnancy before rupture, I should advise its immediate removal by abdominal section, as being more certain and far more safe than the fancy methods of puncturing the cyst and injecting poisonous fluids, or passing through it some kind of galvanic current."

---

For Daniel's Texas Medical Journal.

